# Oncoplastic level II volume displacement surgery for breast cancer: oncological and aesthetic outcomes

**DOI:** 10.1007/s13304-023-01472-0

**Published:** 2023-03-02

**Authors:** Marco Sparavigna, Marco Gipponi, Luca Carmisciano, Simonetta Franchelli, Giulia Atzori, Chiara Cornacchia, Raquel Diaz, Federica Murelli, Francesca Depaoli, Daniele Friedman, Piero Fregatti

**Affiliations:** 1 Breast Surgery Clinic, San Martino Policlinic Hospital, , L.Go R. Benzi, 10, 16132 Genoa, Italy; 2 Department of Internal Medicine, University of Genoa, Genoa, Italy; 3 Department of Health Sciences (DISSAL), Biostatistics Unit, University of Genoa, Genoa, Italy; 4 Department of Surgical Sciences and Integrated Diagnostic (DISC), School of Medicine, University of Genoa, Genoa, Italy

**Keywords:** Oncoplastic breast conserving surgery, Breast cancer, Breast-Q, Volume displacement

## Abstract

Oncoplastic breast-conserving surgery (OBCS) is increasingly used to treat breast cancer with the dual purpose of performing a radical oncological resection while minimizing the risk of post-operative deformities. The aim of the study was to evaluate the patient outcomes after Level II OBCS as regards oncological safety and patient satisfaction. Between 2015 and 2020, a cohort of 109 women consecutively underwent treatment for breast cancer with bilateral oncoplastic breast-conserving volume displacement surgery; patient satisfaction was measured with BREAST-Q questionnaire. The 5-year overall survival and disease-free survival were 97% (95%CI 92, 100) and 94% (95%CI 90, 99), respectively. In two patients (1.8%), mastectomy was finally performed due to margin involvement. The median patient-reported score for “*satisfaction with breast*” (BREAST-Q) was 74/100. Factors associated with a lower aesthetic satisfaction index included: location of tumour in central quadrant (*p* = 0.007); triple negative breast cancer (*p* = 0.045), and re-intervention (*p* = 0.044). OBCS represents a valid option in terms of oncological outcomes for patients otherwise candidate to more extensive breast conserving surgery; the high satisfaction index also suggests a superiority in terms of aesthetic outcomes.

## Introduction

Breast cancer surgical techniques have evolved in patient recovery, oncological safety, and cosmetics toward less invasive approaches [1, 2]. However, although indications for Breast Conserving Surgery (BCS) are more and more increasing, aesthetic results show a great variability with up to 30% of patients reporting unsatisfactory results requiring further surgical correction [3–6]. Several oncological procedures have been developed from pure plastic cosmetic procedures by means of oncoplastic breast conserving surgery (OBCS) with the aim of improving aesthetic results thanks to immediate breast re-shaping. Another key aspect of oncoplastic surgery is the possibility of reducing the rate of positive margins as well as the need of re-excision or mastectomy due to larger excision volumes of lumpectomy [7–9].

Notwithstanding the wide adoption of OBCS procedures, the oncological and cosmetic benefit have not yet been validated in robust studies, mostly as regards their oncologic safety as well as their satisfaction index [1, 10–13]. On these grounds, the oncologic safety as regards local control, disease-free survival (DFS) and overall survival (OS) were assessed in a cohort of 109 consecutive patient undergoing Wide Local Excision (WLE) and Level II volume displacement reconstruction and immediate contralateral breast symmetrisation. Moreover, patient’s satisfaction index was assessed by means of the BREAST-Q patient reported outcome (PRO) and the association between tumour’s or patient’s features and the aesthetic result were computed [14, 15].

## Methods

Between February 2015 and November 2020, a retrospective cohort study was undertaken in patients undergoing OBCS. All patients were consecutively submitted to WLE for breast cancer Level II volume displacement reconstruction and immediate contralateral breast symmetrisation.

Oncoplastic technique was determined by tumour location, tumour size to breast volume ratio, patient’s anatomy, and individual preference. Preoperative drawings were done on the day before surgery to provide guidance to the oncologic procedure thus avoiding any undue skin or glandular excision. According to Clough classification Level II OBCS was chosen since 20–50% of breast volume was to be excised [7]. An intra-operative margin evaluation was always performed; free excision margins required a clear margin according to the rule “*no ink on tumor*” for invasive cancer, or 2 mm for DCIS. Clips were routinely placed at the cardinal points of the tumour bed before gland re-modelling [16].

Clinico-pathological data including demographic information, tumour, treatment and follow-up were recorded into a standardized institutional database. Adjuvant treatment, as well as neo-adjuvant treatment protocol were defined according to evidence-based guidelines of Breast Cancer management edited by the Italian Association of Medical Oncology (AIOM); each case was weekly discussed at the Institutional Breast Disease Management Team. Patients were checked by annual clinical examination, laboratory, and imaging. Local recurrence was defined as histologically proven recurrent tumour occurring within the same breast or skin envelope.

The patient’s satisfaction index was assessed by means of the validated BREAST-Q questionnaire post-operative reconstruction module; it is a patient reported outcome tool developed to assess satisfaction index and health-related quality of life after different breast cancer surgical procedures. The questionnaire was administered at least one year after surgery and it was scored by means of Q-Score software developed using the Rasch model that gives a score on a 0 to 100 scale, with higher values indicating a greater satisfaction index [14, 15].

The primary endpoint was oncological outcome (loco-regional and/or distant disease control); DFS and OS were computed from the date of surgery. Overall survival curves were obtained by means of Kaplan–Meier method. The secondary endpoint of the study was represented by the patient’s satisfaction index after the OBCS procedure. Univariate analyses to assess the association between patient’s, tumour’s and surgery’s features and aesthetic results were performed with linear regression using the Rasch transformed Breast-Q score as dependent variable. Beta coefficients, 95% confidence interval (CI) and *p* values were reported. Since only a single Breast-Q measure per patient was available and the time from surgery to questionnaire administration was heterogenous between subjects, we used *Spearman*’s correlation coefficient to evaluate if the average aesthetic outcome had changed at the increasing of time between surgery and data collection. Two-sided *p* values below 0.05 were considered as significant.

## Results

Overall, 134 patients undergoing OBCS procedures were recruited between 2015 and 2020; 19 of them were not included due to missing data. So, 115 patients were contacted by phone and were offered the Breast-Q Test but six of them refused so that the final sample included 109 patients. The mean age of patients was 57.8 years (SD = 10,59); 26 of them (24%) were smokers (Table 1). Mean operative time was 212 min (SD = 56); 79 (72.5%) patients underwent sentinel lymph-node biopsy (SLNB). Axillary lymph node dissection (ALD) was performed in 24 patients (22%) due to intraoperative diagnosis of SLNB macro-metastases; moreover, six patients (5.5%) had immediate ALD due to pre-operative histologic diagnosis of nodal metastasis. As regards the OBCS procedure, an upper pedicle was chosen for the reconstructive part in 36 patients (33,1%) and a lower pedicle in the remaining 73 patients (66,9%) with a mean resected volume equal to 324 g; the nipple-areolar complex (NAC) was amputated and grafted in 36 patients (33%). The mean length of hospitalization was 3.7 days (range: 1 to 9) (Table 2).

**Table 1:** Clinico-pathological features

	Overall
*n*	109
Age, mean (SD)	57.8 (10.6)
Smoker, *N* (%)	26 (24)
Menopause, *N* (%)	71 (65)
Maximum diameter (mm), mean (SD)	
Mammography	27.4 (23.3)
Magnetic resonance	27.6 (20.4)
Sonography	27.5 (23.0)
Multifocal, *N* (%)	37 (34)
Histological type	
IDC	91 (83.5)
DCIS	12 (11.0)
CLI	6 (5.5)
Tumour (T), *N* (%)	
Tis	11 (10)
0	13 (12)
1	44 (40)
2	36 (33)
3	5 (4.5)
Nodes (N), *N* (%)	
N0	75 (69)
N1	24 (22)
N1mic	4 (4)
N2–3	6 (5)
Prognostic biological features, *N* (%)	
ER +	66 (62.9)
PGR +	65 (62.5)
Her2 +	23 (22.5)
Triple negative	15 (14.4)
Neo-adjuvant therapy, *N* (%)	35 (32)
Adjuvant post-operative therapy, *N* (%)	62 (60)
Radiation therapy, *N* (%)	91 (90)

*IDC* invasive ductal carcinoma, *DCIS* ductal carcinoma in situ, *ILC* invasive lobular carcinoma, *ER* estrogen receptor, *PGR* progesterone receptor, *Her2* C-erb-2 oncoprotein positive

**Table 2:** Surgical procedure data

	Overall
Operative time [minutes], mean (SD)	212 (56)
SLB, *N* (%)	79 (72.5)
SLB + ALD, *N* (%)	24 (22.0)
ALD, *N* (%)	6 (5.5)
Pedicle, *N* (%)	
Superior	36 (33.1)
Inferior	73 (66.9)
NAC graft	36 (33.0)
Resected volume [g], mean (SD)	324 (78)
Hospitalization [days], mean (SD)	3.7 (1.3)

*SLB* sentinel lymph node biopsy, *ADL* axillary lymph node dissection, *NAC* nipple-areolar complex

Margin involvement at definitive histology occurred in 3 patients (2.7%) cases; based on residual breast volume two of them underwent areola and nipple-sparing mastectomy (1.8%) while another patient was still amenable to conservative resection. Post-operative complications occurred in 19 patients (17%) but they were all managed by conservative treatment (Table 3).

**Table 3:** Re-operations and post-operative complications

	*N* (%)
Re-operations:	3 (2.7)
Breast conserving surgery	1 (0.9)
Mastectomy	2 (1.8)
Complications:	19(17)
Seroma	7 (6.4)
Infection	3 (2.7)
NAC necrosis	2 (1.8)
Skin ischemia	3 (2.7)
Hematoma	4 (3.6)

**Fig. 1:**
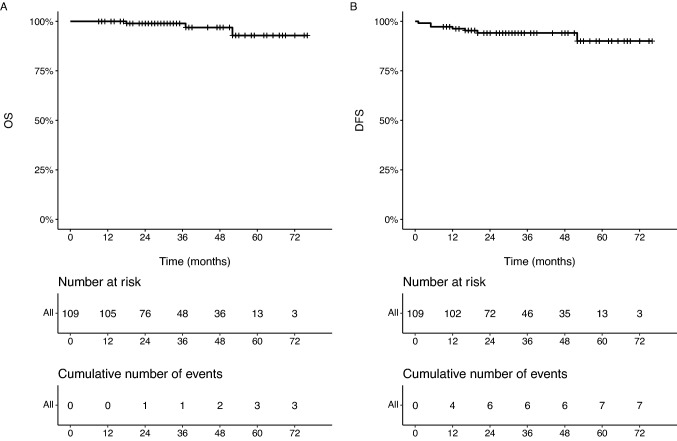
Overall (panel A) and disease free (panel B) survival

As regard as multimodal treatment, 35 patients (32%) underwent preoperative neo-adjuvant therapy; based on definitive histology, 62 patients (60%) underwent adjuvant chemotherapy and 91 patients (90%) underwent radiation therapy (RT). The mean follow-up was 36 months (SD = 17.7). Local recurrence was histologically diagnosed in seven patients (6.4%). Three patients died, two of them having a previous disease relapse; 5 year OS and DFS were 97% (95%CI 92, 100) and 94% (95%CI 90, 99), respectively (Fig. 1).

**Fig. 2:**
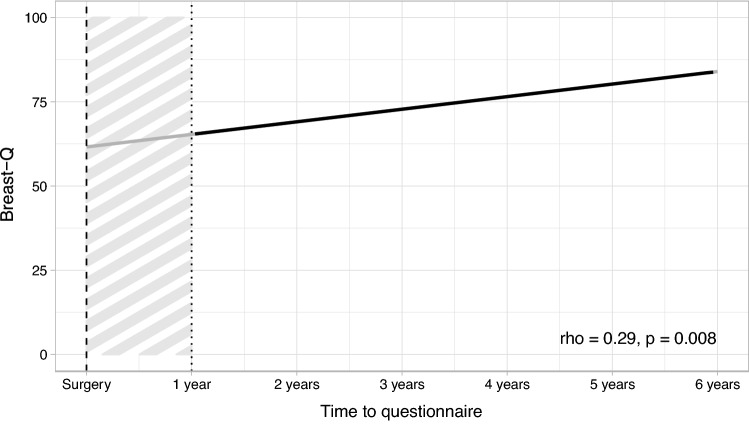
Correlation between average Breast-Q scores and time elapsed from surgery to questionnaire administration. Spearman’s rho = 0.29, *p*-value = 0.008

The mean time elapsed between surgery and questionnaire administration was 36 months (SD = 17.7). Statistical analysis of BREAST-Q questionnaire responses gave an average patient’s satisfaction index equal to 74/100 (range: 63 to 91), the questionnaire response rate was 80%. The predictors of a negative aesthetic satisfaction index were represented by: tumour location (central quadrant) (*p* = 0.007); triple negative breast cancer (*p* = 0.045), and re-intervention (*p* = 0.044) (Table 4). Moreover, a direct correlation between the average satisfaction index and the length of time elapsed from surgery was observed (Spearman’s rho − 0.29; *p* = 0.008) (Fig. 2).

**Table 4:** Univariable linear regression models result

	Beta (95%CI)	*p*
Age (10 year increase)	− 0.5 (− 4.9, 3.9)	*0.815*
Smoke (yes *vs* no)	− 2.2 (− 12.6, 8.3)	*0.681*
T (T3 or T2 *vs* Tis, T0 or T1)	− 0.7 (− 10.4, 9.0)	*0.889*
ALD	0.9 (− 9.0, 10.8)	*0.855*
Histotype (dcis *vs* cdi or cli)	− 11.1 (− 24.8, 2.7)	*0.112*
Focality (multi *vs* mono)	− 0.6 (− 10.1, 8.9)	*0.903*
Central quadrant involvement	− 23.4 (− 40.2, − 6.7)	0.007*
Medium volume removed [× 10]	− 0.07 (− 0.20, 0.07)	*0.332*
Neo-adjuvant	2.1 (− 7.3, 11.5)	*0.657*
Radiotherapy	− 12.0 (− 31.5, 7.5)	*0.225*
Hormone positive	4.1 (− 6.3, 14.4)	*0.436*
HER2 +	8.5 (− 1.9, 18.8)	*0.107*
Triple negative	− 12.5 (− 24.8, − 0.3)	0.045*
Non-surgical complication	− 8.0 (− 19.3, 3.4)	*0.167*
Re-intervention	− 16.4 (− 32.4, − 0.5)	0.044*
Nipple repositioning	− 3.5 (− 13.1, 6.1)	*0.468*

Bold: statistically significant results. Italics: results

Beta coefficients represent an estimate of the average changes in rash transformed Breast-Q score (ranging from 0 to 100) for a change of each characteristic

## Discussion

### Oncologic outcome

The paradigm of BCS is to perform a local radical excision while achieving a satisfactory breast shape; oncoplastic surgery aims at combining the principle of “*free-edge*” resection with the principles of reconstruction for optimizing cosmetic outcomes and minimizing post-operative complications. Oncoplastic procedures includes a wide range of techniques: from simple parenchymal re-arrangement to more complex breast reduction techniques [17, 18].

Oncologic safety would seem to be guaranteed by oncoplastic resection proven the basic principles of local radical resection are respected (“*free-edge*” resection). Although data from randomized controlled trials (RCTs) in OBCS procedures are still lacking, the principles of BCS are firmly rooted in solid evidence. As a matter of fact, large RCTs have confirmed that lumpectomy with post-operative RT gives a lower rate of local recurrence as compared to wide local excision alone [3–19]. Consequently, oncoplastic surgery should give a comparable if not a superior oncological outcome as compared to standard BCS thanks to breast reduction techniques that enables an even larger tumour excision whenever a less than favourable tumour to breast volume ratio does exists, without compromising cosmetics [20–23].

To assess the oncological validity of this procedure we tried, at first, to compare our positive margins rate 2.7% with conventional lumpectomy (range: 9% to 36%). Although apparently favourable, such a comparison may not be correct; in fact, the patient candidate for oncoplastic surgery has specific characteristics in tumour width and glandular volume which represent a relative contraindication for pure BCS [24]. Moreover, in agreement with the prospective series of 101 oncoplastic procedures by *Clough *et al. [25] at the Institute Curie, our findings confirmed that oncoplastic techniques allowed wider resections (324 g *vs* 222 g) which probably explains the lower number of positive margins (2.7% *vs* 10%). In another systematic review by *De La Cruz *et al. [26] regarding the outcome after oncoplastic BCS in 6,011 patients, the positive margin’s rate defined as “*no-ink on tumour*” was 7.8% which compares favourably with our findings, although a direct comparison is not possible due to the broad spectrum of oncoplastic techniques that were herein considered.

The management of patients with a positive margin after an oncoplastic procedure can be really challenging; as a matter of fact, the clear identification of the tumour’s site after extensive glandular re-shaping may not be so easy and this may even require mastectomy. This might explain the rather high conversion to mastectomy rate (CMR) equal to 5.9% and 6.2% that is reported in two other clinical experiences [25, 26]. Our lower CMR (1.8%) might be explained by routine placement of clips at the cardinal points into the tumour’s cavity after tumoral excision coupled with the systematic operative specimen orientation that may aid in addressing a secondary conservative procedure and avoids a more extensive cavity shaving. Finally, as regards oncological outcomes, our recurrence rate of 6.4% compares favourably with literature data (range: 3.1% to 9.4%) [9, 27]. Notably in the review by *Piper *et al. [28] including 1,314 patients undergoing BCS with re-shaping oncoplasty, the recurrence rate was lower (3.1% *vs* 6.4%) even though patients underwent a shorter follow-up (24 *vs* 36 months).

As for SLNB, the most relevant indication is in patients with invasive breast carcinoma; however, as recommended by NCCN guidelines, it is advisable to perform SLB whenever the primary surgical procedure precludes the biopsy at a later time. In our clinical experience, 12 patients with DCIS underwent operation but the extensive mobilization of the remaining gland as well as the location of the excised nodule would not have permitted SLNB at a later time. [29]

As regard surgical complications, our findings compare favourably with literature data; the rates of post-operative infections and NAC necrosis were similar to the findings of [30]: 2.7% *vs* 3.2%, and 1.8% *vs* 1.6%, respectively.

As compared to [26], we experienced an higher rate of axillary seromas (6.4% *vs* 1%) and this could be explained by the higher frequency of DLA (30/109: 27.5%); moreover, we reported a similar rate of post-operative hematomas (3.6% *vs* 2.5%); finally, in 3 out of 109 patients (2.7%) skin ischemia (that is, delayed wound healing without tissue necrosis, not requiring local excision) did occur, comparing favourably with literature data: 2.7% *vs* 2.2%, respectively.

### Patient’s satisfaction index

Although local disease control is the mainstay of breast cancer surgery, it should not affect the aesthetic outcome. In this view, great care should be devoted to the patient’s perception of the result of treatment by means of validated instruments, such as the Patient Reported Outcome Questionnaires. Since its proposal in 2009, the Breast-Q has proved to be highly effective and reliable in the patient satisfaction survey; moreover, a recent consensus recommends documenting the benefits of OBCS by means of the BREAST-q [14, 31]. Efforts have been made to assess if OBCS is comparable to other standard of treatments (BCS and mastectomy with or without reconstruction) in terms of aesthetic outcomes.

Literature data seem to suggest the superiority of OBCS over mastectomy or mastectomy with reconstruction [32, 33]. *Bazzarelli *et al. [15] reported a median patient score of "*satisfaction with breast*" after OBCS and mastectomy equal to 75/100 and 68/100, respectively. *Gardfjell *et al. [34] compared a cohort of 144 patients treated with OBCS Level II volume displacement with two groups of BCS with a median patient-reported score for “*satisfaction with breast*” item of BREAST-q equal to 74/100, 68/100, and 66/100, respectively [35, 36]. These data compare favourably with our findings in the domain "*satisfaction with breast*" that was equal to 74/100.

**Fig. 3:**
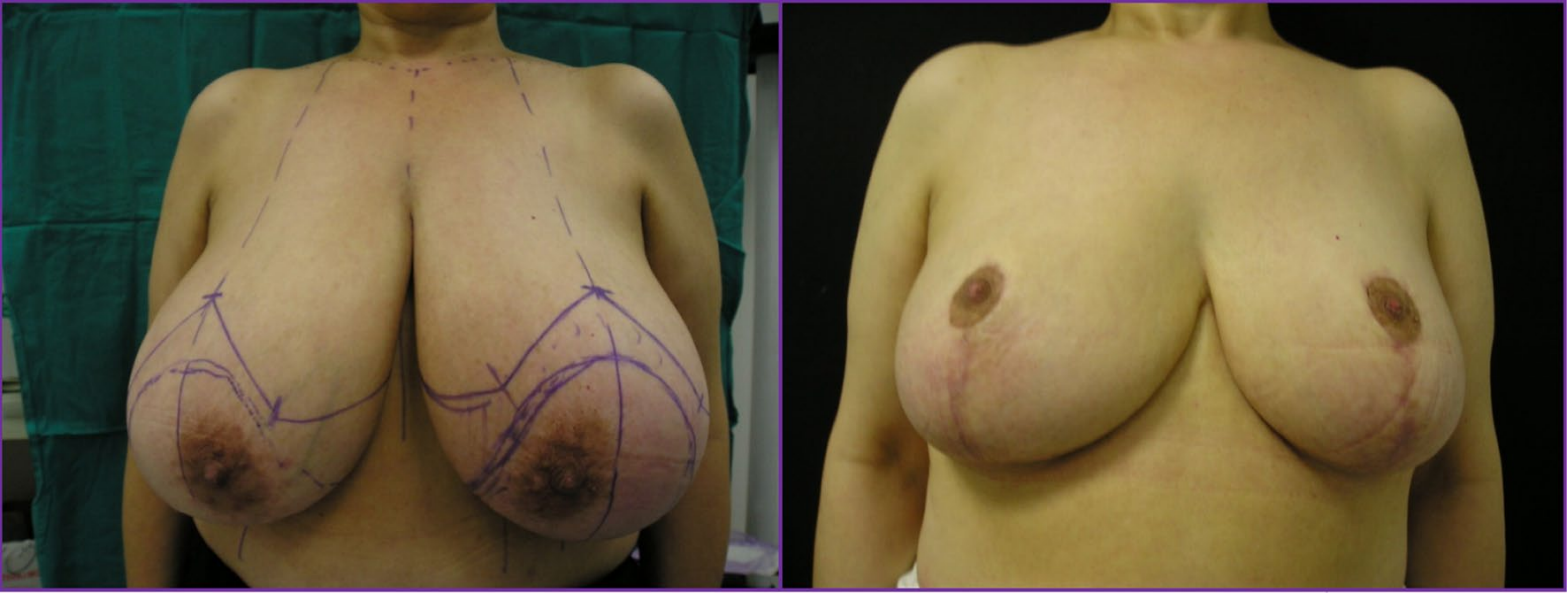
On the left patient affected by breast cancer in the upper quadrants of the left breast with pre-operative markings and subjected to OBCS with inferior pedicle. On the right post-operative results at 3 years.

**Fig. 4:**
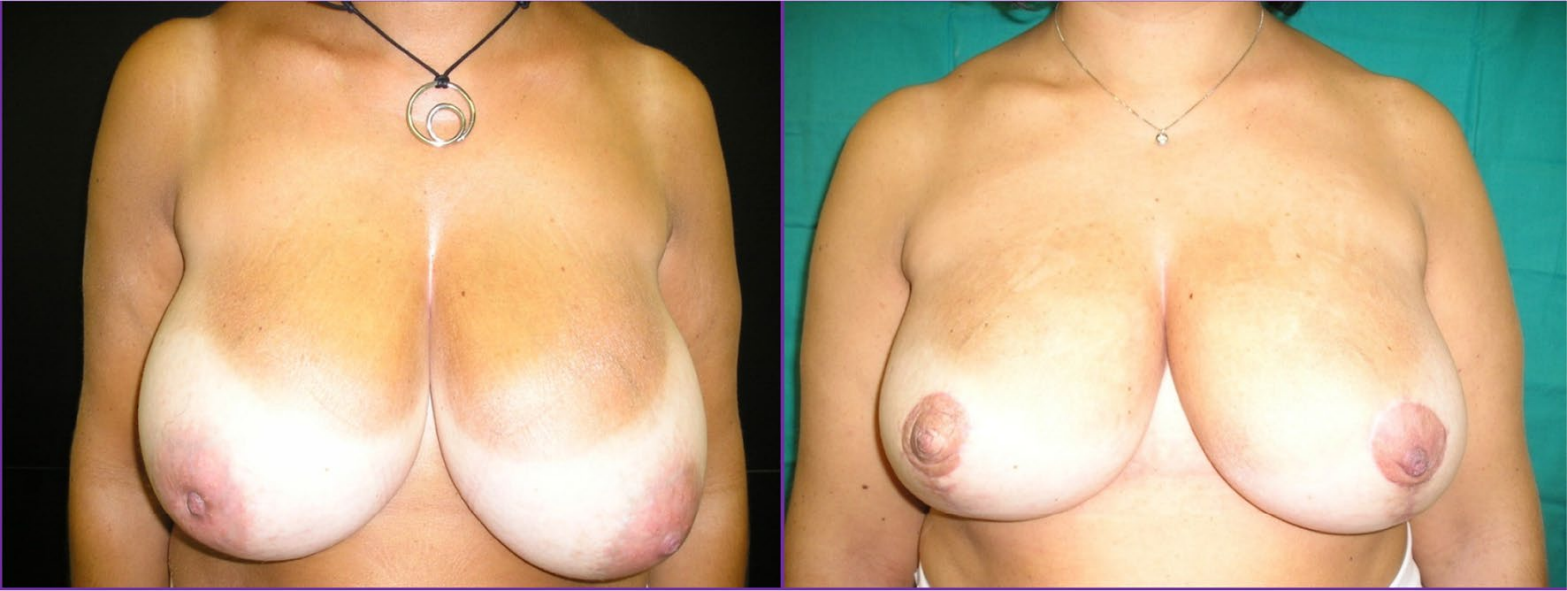
On the left pre-operative imagine of a patient affected by breast cancer in the central quadrant of the right breast and subjected to OBCS with superior pedicle. On the right post-operative image at 4aa

Finally, risk factors usually associated with a lower aesthetic result were not confirmed in our experience; so, for instance, neither the focality nor the medium excised volume showed a statistically significance at univariate analysis. Conversely, quadrant involvement, triple negative biology, and re-intervention were correlated with a decreased aesthetic patient perception. The multiple logistic regression model was not performed due to the rather low number of patients in different subgroups so that further studies are required for evaluating risk factors in larger groups of patients. Worth of noting, an interesting behaviour of time-related satisfaction was observed because the satisfaction index was likely to increase as time elapsed from the procedure, with a weak but significant correlation at Spearman's rho -0.29 (*p* = 0.008). Since the test was given at least one year after surgery, the questionnaire may have been affected by a recall bias (Figs. 3, 4). A similar trend over time was also observed by *Nelson *et al. [37] who demonstrated an improvement in the aesthetic perception of patients undergoing autologous breast reconstruction over 5 years (68.25 *vs* 79.65). As also suggested by *Acea-Nebril *et al. [38], a plurality of factors contribute to the evaluation of aesthetic satisfaction: the length and psychological fatigue of the therapeutic process; the ability to adapt to a new body-image, and the interaction with the medical team. In our view, as time elapses from the conclusion of the treatment planning various factors are likely to positively concur; the physiological improvement of scars; a greater patient’s acceptance of the new physical image and, last but not least, a greater confidence in a positive oncologic outcome.

## Conclusions

Oncoplastic procedures give a great advantage in the management of breast cancer patients thanks to more satisfactory aesthetic results with higher patient’s satisfaction index coupled with a more than acceptable oncological safety; as a matter of fact, a wider removal of breast tissue can be accomplished and this may reduce re-excision and mastectomy rates.
